# Socio-economic and demographic determinants of tobacco use in Kenya: findings from the Kenya Demographic and Health Survey 2014

**DOI:** 10.11604/pamj.2018.30.166.14771

**Published:** 2018-06-25

**Authors:** Peter Magati, Jeffrey Drope, Leopold Mureithi, Raphael Lencucha

**Affiliations:** 1 School of Economics, University of Nairobi, Kenya; 2 Economic and Health Policy Research, American Cancer Society, Inc, 250 Williams Street, Atlanta, GA 30303, USA; 3 Faculty of Medicine, McGill University, School of Physical and Occupational Therapy, Montreal, Quebec, Canada

**Keywords:** Tobacco use, prevalence, tobacco control

## Abstract

**Introduction:**

Every year, more than 6,000 Kenyans die of tobacco related diseases (79 men and 37 women die per week), while more than 220,000 children and more than 2,737,000 adults continue to use tobacco each day. Some suggest that these numbers will rise without concerted efforts to strengthen the implementation of tobacco control measures. To date, there remains much to be learned about what contributes to tobacco consumption in Kenya. This study analyses the socio-economic and demographic determinants of tobacco use in Kenya.

**Methods:**

To analyze the determinants of tobacco use in Kenya, this study uses the 2014 Kenya Demographic and Health Survey. A logistic regression is used to estimate the probability of an individual smoking, given a set of socio-economic and demographic characteristics.

**Results:**

Results suggest that the overall smoking and smokeless prevalence rate is 17.3% and 3.10% respectively among men. Women have low rates with smoking and smokeless prevalence standing at 0.18% and 0.93% respectively. However, for both genders, tobacco use is influenced by age, marital status, residence, region, educational status and gender.

**Conclusion:**

Socio-economic, demographic and geographic disparities on tobacco use should be explored in order to ensure prudent allocation of resources used for tobacco control initiatives. Allocation of resources for tobacco control including monitoring advertisements, sales to underage persons and general distribution of human resource for tobacco control should be based on socio-economic and demographic dynamics.

## Introduction

Tobacco use is one of the leading causes of death globally, accounting directly for nearly 6 million deaths annually and approximately 4% of total disease worldwide-behind only childhood underweight, unsafe sex and blood pressure [1]. It is expected that by 2030, tobacco use will produce the highest burden of premature mortality and disability in the world compared to other health risk factors with low and medium income countries being more affected by this burden than high income countries [2]. In Kenya, every year, more than 6,000 people die of tobacco related diseases, while more than 220,000 children and more than 2,737,000 adults continue to use tobacco each day. Additionally, 79 men and 37 women are killed each week as a result of tobacco use [3]. Kenya actively participated in the negotiations of the WHO Framework Convention on Tobacco Control (FCTC) and in 2004 was one of the first wave of countries to ratify the treaty. In addition, the Tobacco Control Act (TCA) was enacted in 2007 to control the production, manufacture, sale, labelling, advertising, promotion and sponsorship of tobacco products and the regulations gazette in 2016 [4-6].

Despite the passage of the TCA 2007 which institutes a ban on cigarette advertisement, sale of cigarettes to minors, sale of single stick cigarettes to consumers and smoking in public areas, data suggests that tobacco use is still high when compared to countries in sub-Saharan Africa. GATS data shows that the prevalence rate of smoking in Kenya is high when compared to other African countries with 11.6% or 2.5 million Kenyan adults consuming tobacco (19.1% men and 4.5% female). In Uganda the prevalence rate stands at 7.9% (11.6% men and 4.6% women); Nigeria at 5.6% (10.0% men and 1.0% women); Senegal at 6.0% (11.0% men and 1.2% women) while Cameroun is at 8.9% (13.9% men and 4.3% women) [7-9]. The economic costs and the implications for the health and quality of life from these relatively high levels of consumption in Kenya are great. The need to strengthen tobacco control efforts is further punctuated by the ever-present tobacco industry who is actively seeking to expand its market in Kenya [10,11]. In Kenya, while a national plan has been developed to combat tobacco use e.g. Tobacco Control Action Plan 2010-2015 and the Strategy for Prevention and Control of NCDs 2015-2020, little is known about the demographic characteristics of tobacco consumption and the variables associated with this consumption. This data is needed to accurately track consumption trends, including the impact of tobacco control policies over time and the role of socioeconomic characteristics in influencing people to consume tobacco products. Understanding these determinants and factors is also important when coming up with intervention policies as scarce resources will be prudently directed towards the factor and socio-economic dynamic with the highest influence. The objective of this paper is to identify and analyze the socio-economic and demographic determinants of tobacco use in Kenya.

## Methods

**Kenya demographic and health survey**: This paper uses the Kenya Demographic and Health Survey (KDHS) 2014 data to analyze the socioeconomic and demographic determinants of tobacco use, focusing on the different determinants of smoking and smokeless tobacco consumption. The KDHS mostly contained questions on maternal and child health issues, but it also collected information on tobacco use. KDHS is a nationally representative household survey that interviewed 36,430 households (13,914 in urban areas and 22,516 in rural areas). The study included a total of 12,819 men and 31,079 women between the ages of 15-54 years from 400 sample points (clusters) in Kenya.

**Data analysis**: This paper adopts a logistic regression model where the dependent variable is a set of binary indicators because either an individual consumes or does not consume tobacco [12,13]. The model therefore constructs a binary logistic model to estimate the probability of a binary response based on a set of predictor variables as is seen for equation 1.

Pr(*y=*1|*x*)=*xβ*+*ε*

Where: dependent variable y-binary indicator for an individual's smoking status, 1 if the individual smokes and 0 if otherwise The set of independent variables x include age group, place of residence (rural or urban), gender, marital status (never married, living together, married, widowed/divorced/separated), county/region, ethnicity, highest level of education (no education/preschool, primary, secondary or higher education), occupation (whether unemployed, agriculture, service, casual labourer) whether the person is the head of the household, wealth (based on asset index calculated by the DHS) health status, perception to smoking and whether the person is staying at his/her permanent home at the time of the interview. The study however proposes to confirm whether there is multicollinearity between wealth and education and if it exists, incorporate education as part of independent variables and wealth (using asset index in the descriptive). Because this model focuses on socio-economic determinants, it does not include price as part of the independent variables for two reasons. First, the KDHS survey that is used does not collect the prices of cigarettes. Secondly, the price of cigarettes does not vary significantly across the country in a given cross section because of the uniform taxes employed in the cigarettes. Further, the survey is collected within one period of time so variation as a result to variation in time is not expected.

## Results

**Descriptive statistics**: The study included a total of 12,819 men and 31,079 women. 62.35% of the respondents resided in rural areas while 37.65% resided in urban areas. The unemployment rate among the male and female respondents is high at 21.67% and 45.67% respectively. The agricultural sector employs 29.08% of male respondents and 26.17% of female respondents with the majority of the male respondents being employed in the service-manual sector (35.09%). Educational attainment is generally the same with 51.10% of male respondents and 50.24% of female respondents indicating that the highest level of educational attainment was primary level. Also, 31.69% of men and 27.66% of female respondents had secondary schooling as highest level while 11.24% of men and 8.65% of female respondents had higher education as highest education level.

**Tobacco use prevalence**: Among the male DHS respondents, 17.30% and 3.10% indicated using smoking and smokeless tobacco respectively (table 1). The prevalence is low among male respondents between ages 15-19 years, with smoking at 2.06% and smokeless at 0.39%. However the prevalence increases as the age bracket changes peaking with those at the age 45-49 years having the highest prevalence at 28.49%. Similarly, smokeless tobacco use increases with age peaking at 5.79% with those between 35-39 years before reduction of between 4.34% and 4.36% for those between 40-49 years and increasing again to 5.71% for those between 50-54 years. Smoking prevalence is seen to be similar for both urban and rural areas with urban areas having a slightly higher rate. Similar trend is seen for smokeless tobacco use among males but with rural areas being slightly higher. Tobacco use among men also varies with educational levels. Those with no education have a higher consumption of smokeless tobacco at 20.10% and this reduces as educational level increase with those having higher education having a prevalence of 0.56%. However, while the smoking prevalence is 15.67% for those with no education, the number increases to 21.76% for those with primary schooling but the prevalence rate drops as educational levels increase reaching 10.48% for those with higher education qualification. Male smoking prevalence is highest among those who are employed in the agricultural sector and service-manual sectors at 21.52% and 21.62% respectively while those in the non-manual sector have a prevalence rate of 12.76%. Male respondents that are unemployed have the lowest prevalence rate of 2.31%. Similar trend is seen for smokeless tobacco with the unemployed having the lowest rate of 0.83% while those in the agricultural and service-manual the highest at between 4.63% and 3.92% respectively.

**Table 1:** Male and female smoking and smokeless use in Kenya

	Men					Women		
Characteristics	N	Smoking Prevalence	Smokeless Prevalence	Dual Use	N	Smoking Prevalence	Smokeless Prevalence	Dual Use
*Total*	12,819	17.30%	3.10%	0.87%	31,079	0.18%	0.93%	0.02%
***Age Group***								
15-19	2,811	2.06%	0.39%	0.14%	6,078	0.07%	0.20%	n/a
20-24	1,981	10.45%	1.67%	0.50%	5,405	0.13%	0.68%	0.02%
25-29	1,942	18.95%	2.88%	0.82%	5,939	0.25%	0.88%	0.03%
30-34	1,701	25.22%	4.35%	1.35%	4,452	0.29%	0.97%	0.02%
35-39	1,486	25.17%	5.79%	1.62%	3,868	0.13%	1.22%	0.03%
40-44	1,198	25.21%	4.34%	1.42%	2,986	0.13%	1.44%	0.03%
45-49	895	28.49%	4.36%	0.78%	2,351	0.30%	2.38%	n/a
50-54	805	27.95%	5.71%	1.24%	-	n/a	n/a	n/a
***Marital Status***								
Never Married	5,400	8.59%	1.20%	0.44%	8,575	0.13%	0.22%	0.02%
Living Together	254	18.11%	9.45%	1.97%	1,285	0.39%	2.18%	0.08%
Married	6,439	21.29%	4.13%	1.03%	17,751	0.14%	1.00%	n/a
Widowed/Divorced/Separated	726	46.42%	5.79%	2.20%	3,468	0.43%	1.90%	0.09%
***Region***								
Coast	1,598	22.65%	2.00%	0.63%	3,902	0.33%	0.87%	n/a
North Eastern	624	9.29%	3.21%	1.92%	1,664	n/a	0.06%	n/a
Eastern	2,302	29.37%	4.26%	1.26%	5,247	0.10%	0.88%	0.02%
Central	1,370	26.72%	1.24%	0.51%	3,114	0.16%	0.03%	0.03%
Rift Valley	3,673	12.01%	5.45%	1.17%	9,059	0.22%	2.29%	0.03%
Western	1,217	11.01%	1.56%	0.41%	2,840	0.11%	n/a	n/a
Nyanza	1,649	7.22%	0.36%	0.12%	4,254	0.05%	n/a	n/a
Nairobi	386	16.06%	1.30%	0.78%	999	0.70%	0.10%	0.10%
***Residence***								
Urban	4,915	17.84%	1.75%	0.71%	11,614	0.25%	0.32%	0.02%
Rural	7,904	16.97%	3.93%	0.96%	19,465	0.13%	1.30%	0.02%
***Education Level***								
No Education	766	15.67%	20.10%	2.74%	4,183	0.26%	6.22%	0.05%
Primary	6,550	21.76%	2.89%	1.02%	15,613	0.15%	0.17%	0.01%
Secondary	4,062	12.85%	1.13%	0.47%	8,595	0.14%	0.01%	n/a
High Education	1,441	10.48%	0.56%	0.28%	2,688	0.30%	0.07%	0.07%
***Wealth Quantiles***								
Poorest	2,683	18.67%	9.21%	1.75%	7262	0.17%	3.77%	0.06%
Poorer	2,578	20.21%	2.13%	0.85%	5970	0.13%	0.15%	n/a
Middle	2,636	18.32%	1.67%	0.68%	5946	0.12%	0.07%	n/a
Richer	2,758	15.55%	1.31%	0.73%	5958	0.18%	0.02%	n/a
Richest	2,164	13.12%	0.69%	0.18%	5943	0.29%	0.03%	0.03%
***Occupation***								
Unemployed	2,294	2.31%	0.83%	0.26%	5630	0.44%	2.42%	0.04%
Agriculture	3,109	21.52%	4.63%	1.19%	3226	0.03%	0.99%	n/a
Service-Manual	3,751	21.62%	3.92%	1.12%	2103	0.52%	2.09%	0.10%
Non-Manual	1,536	12.76%	0.91%	0.26%	1369	0.37%	0.80%	0.07%

Counties that fall in the Eastern region have the highest prevalence rates for male smokers at 29.37%, followed by the Central counties where the smoking prevalence is 26.72%, Coastal region at 22.65%, Nairobi has a prevalence of 16.06% while Rift valley and Western regions have prevalence of 12.01% and 11.01% respectively. Smokeless tobacco among men is high in counties in Rift valley, Eastern and North Eastern regions having a prevalence of 5.45%, 4.26% and 3.21% respectively. Kenya's constitution created a two tier governance structure which share health responsibilities whereby the central/national government is responsible for policy formulation on health issues, while the implementation lies with the county governments. The results suggests that smoking prevalence rates for men are high in Meru, Isiolo, Kitui Embu and Kirinyaga counties with all these recording a prevalence rate of above 30% with Meru particularly high at 38%. However Turkana, Samburu and Marsabit Counties have very high smokeless prevalence rates of 31.82%, 23.31% and 22.58% respectively. Among the female DHS respondents, Smoking prevalence is 0.18% while the smokeless tobacco at 0.93% (Table 1). Although the prevalence was low compared to men, similar patterns by demographic characteristics were observed. For instance while the consumption of both increases as the respondents get older. However, while the prevalence of smoking increases from 0.07% at 15-19 years, it seems to peak at 0.29% at age 30-34 years before dropping to 0.13% between 35-49 years before peaking at the highest rate recorded at 0.3%. This is different from the prevalence of smokeless tobacco that increases progressively with age from 0.2% at the age of 15-19 years to 2.38% at the age 45-49 years. Female respondents with no education have the highest prevalence of smokeless tobacco at 6.22% with the prevalence rate of smokeless tobacco reducing as the educational level increase. For smoking prevalence, the rate is highest and similar for those with highest educational level 0.30%.

Results also suggest that while tobacco use among women is still low, prevalence varies across regions. Regionally, Nairobi County has the highest smoking prevalence among women at 0.7% followed by the coastal region at 0.33%, the Rift valley counties at 0.22% and counties in the Central region at 0.16%. Only Eastern and Western regions have prevalence rates of higher that 0.1% in addition to Coastal, Rift Valley and Central regions at 0.11% 0.1% respectively. When prevalence is analysed per county, Turkana and Mombasa County recorded the highest smoking prevalence rate at 1.75% and 1.17% respectively Smokeless tobacco use is however highest among women in Rift Valley with a prevalence rate of 2.29%. This is followed by Eastern at 0.88%, Western at 0.87% and Nairobi at 0.1%. Other regions have lower smokeless prevalence among women. Specifically, Samburu and Turkana have the highest smokeless prevalence rates at 16.58% and 14.59% respectively. Relatively high rates compared to the national average is recorded in Marsabit at 5.39%, Kilifi at 2.31%, Laikipia at 2.5%, Isiolo at 1.98% and Kwale County at 1.34%.

**Determinants of tobacco use**: Table 2 displays multivariable results for smoking and smokeless tobacco use stratified by gender. Results suggest that smoking prevalence among men increases significantly with age with men ≥20 years five to thirteen times likely to smoke. For example, men between 20 years and 30 years are five to nine times more likely to smoke while those between 30 and 54 years are twelve to thirteen times likely to smoke tobacco. On the other hand, prevalence of Men using smokeless is likely to be between five and eight times for men ≥20 years. The Male smoking prevalence between the singles and those who lived together with a partner was likely to be the same. However, married men were 15% less likely to smoke while those who are divorced, separated or widowed were 1.4 times likely to smoke tobacco. Results however also suggest that while married men's prevalence of smokeless tobacco was low, those whose who lived with a partner were twice as likely to use smokeless tobacco while those who are divorced, separated or widowed were 1.5 times as likely to use smokeless tobacco. Despite the low rates of smoking and smokeless tobacco use, smoking patterns among females was robust. Women in the age bracket of 20-30 are as much as two times more likely to use smokeless tobacco compared to those 15-19 years while those aged 45-49 years are 3.8 times more likely to smoke compared to those aged 15-19 years. Smoking prevalence also increases with age albeit with mixed results. We see that there is a probability that those aged between 20 to 30 years are between two and four times likely to smoke cigarettes. This number reduces for those aged between 30 and 40 years because the probability of women using cigarettes in this age is 92% more likely to use cigarettes but for those above 40 years, there is a huge surge with women up to 4 times likely to use cigarettes. Married women are the least likely to use both smoking and smokeless tobacco products. Results suggest that those who lived together with a partner were 46% more likely to use smoking tobacco and 48% more likely to use smokeless tobacco. However, when the women got married, the probability of smoking reduces by 41% while that of using smokeless tobacco reduces by 26%.

**Table 2:** Adjusted prevalence ratios and 95% confidence intervals of smoking and smokeless tobacco use, male DHS respondents

	Male Smoking		Male Smokeless		Female Smoking		Female Smokeless	
	PR	95% CI	PR	95% CI	PR	95% CI	PR	95% CI
**Age Group**								
15-19	1		1		1		1	
20-24	5.58	(4.19-7.42)	4.98	(2.11-8.28)	1.82	(0.50-6.63)	1.75	(0.87-3.51)
25-29	9.96	(7.54-13.15)	5.8	(2.94-11.44)	3.7	(1.06-12.87)	1.96	(0.98-3.91)
30-34	12.75	(9.61-16.91)	7.3	(3.65-14.58)	4.42	(1.22-15.96)	2.36	(1.17-4.76)
35-39	12.94	(9.71-17.25)	9.48	(4.74-18.99)	1.92	(0.44-8.29)	2.67	(1.33-5.38)
40-44	12.93	(9.68-17.28)	7.81	(3.83-15.92)	1.92	(0.41-8.94)	2.7	(1.33-5.47)
45-49	13.74	(10.27-18.40)	8.17	(3.96-16.87)	4.12	(1.01-17.76)	3.8	(1.90-7.59)
50-54	13.37	(9.96-17.94)	8.3	(4.05-16.99)				
**Marital Status**								
Never-married	1		1		1		1	
Living-together	0.98	(0.75-1.29)	1.99	(1.29-3.07)	1.46	(0.46-4.62)	1.48	(0.81-2.72)
Married	0.85	(0.76-0.96)	1.03	(0.74-1.44)	0.59	(0.25-1.38)	0.74	(0.44-1.26)
Widowed/divorced/separated	1.44	(1.26-1.63)	1.57	(1.04-2.38)	1.66	(0.66-4.21)	1.2	(0.68-2.12)
**Region**								
Coast	1.79	(1.59-2.02)	0.39	(0.28-0.56)	1.43	(0.70-2.90)	0.33	(0.23-0.47)
North Eastern	1	(0.77-1.30)	0.25	(0.16-0.38)	0	(0.00-4.60)	0.01	(0.00-0.05)
Eastern	2.26	(2.04-2.50)	0.86	(0.68-1.07)	0.46	(0.17-1.22)	0.47	(0.35-0.65)
Central	2	(1.78-2.25)	0.34	(0.21-0.56)	0.73	(0.27-1.98)	0.07	(0.01-0.05)
Rift Valley	1		1		1		1	
Western	0.93	(0.78-1.11)	0.39	(0.25-0.62)	0.53	(0.16-1.81)	0	(0.00-2.60)
Nyanza	0.64	(0.53-0.77)	0.11	(0.05-0.25)	0.26	(0.06-1.10)	0	(0.00-2.50)
Nairobi	1.31	(1.03-1.66)	0.6	(0.24-1.48)	2.4	(0.95-6.06)	0.27	(0.04-1.92)
**Residence**								
Urban	1		1		1		1	
Rural	0.76	(0.90-1.03)	1.61	(1.27-2.04)	0.67	(0.37-1.22)	1.58	(1.12-2.23)
**Highest level of education**								
Secondary+	1		1		1		1	
No-education	1.1	(0.86-1.24)	12.89	(9.39-17.69)	1.92	(0.84-4.38)	159.7	(50.46-505.44)
Primary	1.03	(1.48-1.75)	2.52	(1.86-3.41)	0.98	(0.52-1.83)	5.57	(1.68-18.45)
**Observations**	**12819**		**12819**		**31079**		**31079**	

Due to the relatively lower prevalence rates and wider socio-economic variations especially of population among the younger population i.e age group 15-19 years, this study conducted a multivariate analysis of the adult population (defined as those ≥22 years). The results of the multivariate analysis among adults is shown in Table 3. The occupational status was included in the adult model with results suggesting men in the agricultural sector have the highest likelihood to smoke (PR=1.51, 95% CL 1.07-2.13) followed by those in non-manual occupations (PR=1.49 95% CL 1.06-2.11) while those in the services manual have the lowest likelihood with the PR being 0.95 (95% 0.66-1.36). Similar pattern was followed in smokeless use by men with those in the services manual being less likely to use smokeless tobacco. Unlike previously when young adults were included, men with no education were less likely to smoke (PR=0.74, 95% CL 0.58-0.93) while the PR for men with primary education was 1.33 (95% CL 1.21-1.47). Tobacco use among women was difficult to assess in multivariable analysis given the wide variations of the confidence levels, mainly because of low number of women who reported using tobacco in the survey. However, like the men, results show that tobacco use was positively associated with age while those with no education more likely to smoke (PR= 1.68, 95% CL 0.59-4.7).

**Table 3:** Adjusted prevalence ratios and 95% confidence intervals of smoking and smokeless tobacco use, adult (age 23-59) DHS respondents

	Male Smoking		Male Smokeless		Women Smoking		Women Smokeless	
	PR	95% CI	PR	95% CI	PR	95% CI	PR	95% CI
**Age Group**								
23-29	1		1		1		1	
30-40	1.41	(1.25-1.60)	1.65	(1.20-2.30)	1.45	(0.67-3.12)	1.67	(1.21-2.29)
40-50	1.49	(1.31-1.70)	1.46	(1.02-2.08)	1.73	(0.74-4.04)	1.89	(1.37-2.61)
50-59	1.53	(1.31-1.79)	1.65	(1.11-2.46)				
**Marital Status**								
Never-married	1		1		1		1	
Living-together	0.87	(0.63-1.20)	2.07	(1.17-3.65)	1.7	(0.36-7.96)	1.84	(0.60-5.63)
Married	0.81	(0.71-0.93)	1.44	(0.94-2.22)	0.72	(0.20-2.52)	1.1	(0.38-3.24)
Widowed/divorced/separated	1.35	(1.16-1.57)	1.96	(1.16-3.32)	2.33	(0.62-8.75)	1.77	(0.59-5.32)
**Region**								
Coast	1.7	(1.48-1.96)	0.41	(0.28-0.62)	1.41	(0.63-3.15)	0.37	(0.25-0.55)
North Eastern	1.05	(0.74-1.49)	0.14	(0.06-0.29)	0		0	
Eastern	2.1	(1.86-2.37)	0.9	(0.69-1.17)	0.52	(0.17-1.59)	0.45	(0.31-0.64)
Central	1.95	(1.71-2.22)	0.36	(0.21-0.62)	0.58	(0.13-2.58)	0.13	(0.01-0.91)
Rift Valley	1		1		1			
Western	1.03	(0.86-1.26)	0.45	(0.27-0.75)	0.74	(0.21-2.59)	0	0
Nyanza	0.63	(0.51-0.78)	0.1	(0.04-0.26)	0.23	(0.03-1.72)	0	0
Nairobi	1.36	(1.02-1.82)	0.98	(0.39-2.46)	1.86	(0.50-6.88)	0	0
**Residence**								
Urban	1		1		1			
Rural	0.95	(0.87-1.04)	1.73	(1.27-2.36)	0.86	(0.43-1.71)	1.91	(1.28-2.84)
**Highest level of education**								
Secondary+	1		1		1			
No-education	0.74	(0.58-0.93)	8.97	(6.30-12.79)	1.68	(0.59-4.76)	1.25E+11	(0.00-8.90E+187)
Primary	1.33	(1.21-1.47)	1.7	(1.21-2.36)	1.46	(0.62-3.45)	4.96E+09	(0.00-3.50E+186)
**Occupation**								
Unemployed	1		1		1		1	
Agriculture	1.51	(1.07-2.13)	0.58	(0.36-0.93)	0.04	(0.01-0.31)	0.58	(0.40-0.84)
Non-manual	1.49	(1.06-2.11)	0.64	(0.40-1.00)	0.58	(0.27-1.24)	1.32	(1.00-1.75)
Service-manual	0.95	(0.66-1.36)	0.21	(0.11-0.43)	0.37	(0.12-1.17)	0.75	(0.38-1.49)
**Observations**	**7023**		**7023**		**8249**		**8249**	

## Discussion

Results highlight differences and patterns of tobacco use in Kenya. Smoking has a bigger magnitude among men than smokeless tobacco. However, both are consistent as the prevalence is considerably more among older men than the younger ones. The same can be said among women though smokeless tobacco use is higher. This is expected because of the addictive nature of tobacco consistent with other findings that conclude that soon after initiation, nicotine addiction makes withdrawal unpleasant and many find themselves regular smokers [14,15]. Low prevalence among young adults (≤22 years) could also suggest that ban on advertising and increase in tobacco taxes have been effective tools. Older generations have more spending power and have relatively higher prevalence rate because at their time of youth, advertising and branding was allowed making initiation easier. Research shows that initiation usually begins in teenage with tobacco advertisement and other marketing strategies of the industry such as branding leading to tobacco initiation and sustained use among the teenagers [16-18]. It is also important to note that consumption among women remains low across all regions in Kenya. This is a positive starting point for tobacco control proponents. However, this pattern should not be taken for granted nor ignored. Research has found that the tobacco industry actively targets women as part of their effort to expand their market. Strategies have included manufacturing products specifically targeting women [19,20], and marketing tobacco consumption as a symbol of empowerment [21]. In other words, tobacco control efforts must continue to focus on this demographic to prevent a surge consumption. Consumption patterns in the survey follow a logical pattern because educational levels and public awareness are impactful in reducing tobacco use on the more literate population. The results are consistent with a country at the early stages of the tobacco epidemic [22]. High smokeless tobacco use in rural areas with lower incomes is also expected because smokeless tobacco is cheaper and people likely to consume it. Also, the likelihood of having poor and uneducated is higher in rural areas [23,24]. Further, the findings suggest that perhaps smokers who are poor and uneducated live in rural areas.

## Conclusion

Results suggest that tobacco use in Kenya varies socioeconomic, demographic and geographic factors. County governments should ensure that there is a budget component for tobacco control including monitoring of media advertisements and tobacco sales, with allocation dependent on the prevalence rates of tobacco use in the county as opposed to the current format where counties allocate on adhoc basis. There is also need for the national government, through the ministry of education to ensure that the education policy incorporates tobacco control and non-communicable disease. This is particularly important because the Ministry of Education is currently in the process of reviewing the educational curricular and system meaning that such a move could be effective because initiation takes place in high school and addictive nature means use increases with age, suggesting it is difficult to quit smoking. Already, tough measures undertaken in tobacco control as a result of implementing Kenya's tobacco control Act and tobacco control regulations including advertemsent and sale of cigarettes to anyone under 18 years has resulted to reduced smoking among the younger generation which has lived most of their conscious/independent life after the 2007 Tobacco Control Act. This paper recommends that the government, under the stewardship of the ministry of health, continues to strengthen multi-sectoral public policy that establishes and strengthens tobacco control and other NCD risk prevention factors. This will ensure that the World Health Organization (WHO) 2013-2020 action plan for the prevention and control for NCDs which includes 30% reduction in tobacco use prevalence, with countries having 100% smoke free legislations, building capacity of citizens on dangers of tobacco use through evidence based information and raising taxes among other measures.

### What is known about this topic

- Smoking prevalence rates of women and men in Kenya;
- Approximate number of fatalities as a result of tobacco use in Kenya.

### What this study adds

- Determinants and factors influencing tobacco consumption among different population groups;
- Consumption patterns among different age groups and regions.

## Competing interests

The authors declare no competing interests.
